# Post-Activation Performance Enhancement as a Strategy to Improve Bench Press Performance to Volitional Failure

**DOI:** 10.5114/jhk/162958

**Published:** 2023-07-15

**Authors:** Arkaitz Garbisu-Hualde, Laura Gutierrez, Jordan Santos-Concejero

**Affiliations:** 1 Department of Physical Education and Sport, University of the Basque Country UPV/EHU, Vitoria-Gasteiz, Spain.

**Keywords:** muscle hypertrophy, strength training, AMRAP, muscle endurance

## Abstract

Post-Activation Performance Enhancement (PAPE) has been commonly used as a strategy to improve acute force production, although its effects on performance to volitional failure are still unknown. The aim of this study was to analyse the influence of a PAPE protocol on bench press performance in a training set to volitional failure in trained individuals. Fourteen participants with at least two years of resistance training experience (age 24.57 ± 2.7 years; body mass 77.47 ± 12.2 kg; body height 174.21 ± 7.4 cm; medium grip bench press 1 repetition maximum (1RM): 101.6 ± 25.8 kg), of which 14 completed the control protocol and 12 completed the experimental protocol, took part in the study. After a standardised warm-up, participants completed three sessions: 1) a 1RM test for the medium grip bench press, 2) a control condition consisting of a set of the bench press to volitional failure with 80% 1RM (CON), and 3) an experimental condition consisting of a set of the bench press to volitional failure with 80% 1RM after a PAPE protocol (PAPE). The PAPE protocol consisted of a heavy set of one repetition with their 93% 1RM as the conditioning activity. Under the PAPE condition, participants performed significantly more repetitions than under the CON condition (p = 0.008, ES = 0.5, small effect), their last repetition was slower (p = 0.02, ES = 0.52, small effect) and presented a higher velocity loss (p = 0.004, ES = 0.75, moderate effect). These results suggest that a traditional PAPE protocol improves the number of repetitions performed to volitional failure.

## Introduction

Improving sport performance requires correctly combining different aspects through a training program. Several strategies are commonly used to improve acute force production in explosive tasks (McGowan et al., 2015) or exercise volume in tasks to volitional failure (Alves et al., 2019), and Post-Activation Performance Enhancement (PAPE) is one of such strategies. PAPE has been defined as a phenomenon that acutely improves voluntary muscle performance (Blazevich and Babault, 2019). PAPE should not be confused with Post-Activation Potentiation (PAP), which needs electrically induced confirmation (Cuenca-Fernández et al., 2017; MacIntosh et al., 2012). Mechanisms behind PAPE are not completely known, but evidence suggests that it may be due to a combination of the mechanism behind PAP (i.e., myosin light chain phosphorylation) and other mechanisms such as muscle temperature or intramuscular water content (Blazevich and Babault, 2019; Rodrigues et al., 2022). Most commonly, the used conditioning activity (CA) and the exercise where performance enhancement is required are biomechanically similar (i.e., a barbell back squat as a CA for squat jumps), as it is more likely to obtain favourable performance results (Cormier et al., 2022). Regarding rest intervals, various recommendations have been proposed, but in a recent study by Cormier et al. (2022), 5–7 minutes are recommended for strong individuals (≥1.35 kg/kg [1RM kg / body mass in kg] in the barbell bench press) and ≥8 minutes for weaker individuals.

PAPE has gained notorious popularity in power related sports due to its capacity to improve the acute rate of force development using different strategies with different muscle contraction regimes as conditioning stimuli (Krzysztofik et al., 2020a; Skurvydas et al., 2019). Various load protocols, such as optimal power loads (5 repetitions of the back squat with the optimum power load with 5 min rest intervals between repetitions) (Gilbert and Lees, 2005), medium loads (4 repetitions of the back squat with 70% 1RM) (Lowery et al., 2012) and even plyometric contractions (3 sets of 5 repetitions of plyometric push-ups with a 1-min rest interval) (Krzysztofik and Wilk, 2020) can be used. Even more, PAPE has started to be studied as an acute mechanism to improve the total volume performed during a training session (Alves et al., 2019; Krzysztofik et al., 2020b). In a study by Alves et al. (2019), a PAP protocol consisting of 3 sets of 1 repetition of the bench press exercise at 90% 1RM showed a performance enhancement in a task to volitional failure in the first two sets compared to a control condition. On the other hand, results by Krzysztofik et al. (2020b) showed no improvement in the number of performed repetitions between PAPE (3 sets of 3 repetitions of the bench press at 85% 1RM) and control conditions, but they found a higher time under tension for the PAPE condition. Evidence so far seems contradictory and scarce, thus, the possible role of PAPE to increase training volume is yet to be elucidated.

Velocity-based training is a well-known method to quantify a strength training stimulus (González-Badillo et al., 2011; Martínez-Cava et al., 2020). Previous research has suggested that PAPE can improve skeletal muscle shortening velocity due to improved Ca^2+^ sensitivity (Boullosa et al., 2018). This results in increased propulsive velocity (Blazevich and Babault, 2019), which may improve the performance of all repetitions in a set to volitional failure, helping the athlete to complete more repetitions at the same intensity. As PAPE has previously demonstrated to improve performance in a task to volitional failure (Alves et al., 2019), the question arises whether this improvement is related to a greater velocity loss between the first and the last performed repetition during a set of the bench press exercise.

PAPE is especially useful in sport performance during submaximal loaded tasks such as sprinting (Iacono et al., 2018), jumping (Lowery et al., 2012) and throwing (Krzysztofik et al., 2022), possibly due to a greater sensitivity to submaximal Ca^2+^ concentrations (Blazevich and Babault, 2019). As most studies show improvements in short-lasting activities, it would be of great interest to analyse what happens when work capacity is tested in a submaximal loaded task, considering the number of repetitions performed until volitional failure. The barbell bench press, one of the most popular exercises for upper body strength and one of the three main lifts in powerlifting competitions (Gomo and Tillaar, 2016), appears to be an interesting choice to answer these questions. Thus, this study aimed to analyse the influence of a traditional PAPE protocol on performance in a set at 80% 1RM, total work performed and last repetition kinematics in a training set to volitional failure in the bench press exercise. Our hypothesis was that, as PAPE protocols are effective in submaximal loads, the number of performed repetitions would be higher under the PAPE condition.

## Methods

### Participants

Fourteen participants (age 24.57 ± 2.7 years; body mass 77.47 ± 12.2 kg; body height 174.21 ± 7.4 cm; medium grip bench press 1 repetition maximum (1RM) 101.6 ± 25.8 kg) with at least 2 years of resistance training experience voluntarily took part in the study. Participants were required to meet the following inclusion criteria: 1) males between the age of 18 and 40 years; 2) lack of musculoskeletal disorders or injury in the previous 6 months; 3) resistance training experience, defined as consistently lifting weights at least 3 times per week for a minimum of 2 years. Fourteen participants met the inclusion criteria and were recruited for the study, from which a total of 14 completed the control protocol and 12 participants completed the experimental protocol. Written informed consent was obtained from each participant after a thorough explanation of the testing protocol, the possible risks involved, and the right to terminate participation at will. The study was conducted according to the Declaration of Helsinki, and the Institutional Review Board of the University of the Basque Country (UPV/EHU) (Ref. CEISH 117/2019).

### Design and Procedures

Participants visited the laboratory on three separated occasions. Prior to every experimental session, participants performed a standardised warm-up consisting of 5-min cycling on a cycloergometer and bench press sets including 10 repetitions with 40% 1RM, four repetitions with 60% 1RM, two repetitions with 70% 1RM, and one repetition with 80% 1RM, as described elsewhere (Krzysztofik et al., 2020a). During the first visit, participants underwent a 1RM test for the medium grip bench press. The 1RM test was performed in the free weight bench press and grip width was set at 1.4 times biacromial distance as described elsewhere (Larsen et al., 2021). In every session, participants were requested to perform a 2-s tempo descent followed by a 1-s pause on the chest (using a metronome at 60 beats per minute) to standardise the repetitions (Krzysztofik et al., 2020a). The 1RM was defined as the highest load lifted by the participant without any compensatory movement only if the participant completed the pause on the chest properly. When an attempt was successful, the next attempt was performed evaluating the reported mean propulsive velocity by the linear encoder T-Force Dynamic Measurement System (ERGOTECH, Murcia, Spain) (Martínez-Cava et al., 2020).

After 72 hours, participants were randomly assigned (www.random.org) to a PAPE (experimental condition) or a CON (control condition) group. The second and third experimental sessions were scheduled 72 hours apart to allow full recovery, and at the same time of the day. During the CON protocol, participants underwent a standardised warm-up, and 4 min after the last approximation set, they performed repetitions until concentric volitional failure with their 80% 1RM. During the PAPE protocol, participants underwent the same standardised warm-up, but they performed a heavy set of one repetition with their 93% 1RM as a conditioning activity (Garbisu-Hualde and Santos-Concejero, 2021) and then were asked for their rate of perceived exertion (RPE). After the conditioning activity, participants rested for 6 min before performing repetitions until concentric volitional failure with their 80% 1RM. The conditioning activity settings (rest interval and the protocol used) were based on the participants´ strength level. This configuration was chosen because participants´ strength levels (1.31 kg/kg) were closer to high strength levels reported by Cormier et al. (2022) (≥1.35 kg/kg [1RM kg/body mass in kg] in the barbell bench press). Participants performed a 2-s tempo descent followed by a 1-s pause on the chest (using a metronome set at 60 beats per minute) during all repetitions, to avoid compensatory movements and not to alter the obtained results. Mean propulsive velocity of the lifts was measured with the linear encoder T-Force Dynamic Measurement System. For the third session, participants changed the conditions. Participants were instructed to lift the barbell as fast as possible during the ascent of all testing lifts, and were requested not to perform any exhausting activity 48 hours prior to the intervention. They were also requested not to consume caffeine or any other stimulant prior to testing.

### Statistical Analysis

Data are presented as means ± SD. All variables presented a normal distribution according to the Shapiro-Wilk test. A paired *t*-test was performed to compare the experimental and the control condition. Additionally, the magnitude of differences of effect sizes (ES) was calculated using Cohen’s *d* (Cohen, 1988) and interpreted as small (>0.2 and <0.6), moderate (≥0.6 and <1.2) and large (≥1.2 and <2) or very large (≥2) according to the scale proposed by Hopkins et al. (2009). All statistical analyses were performed using Prism 9 for Mac. Significance for all analyses was set at *p* < 0.05.

## Results

**Figure 1:**
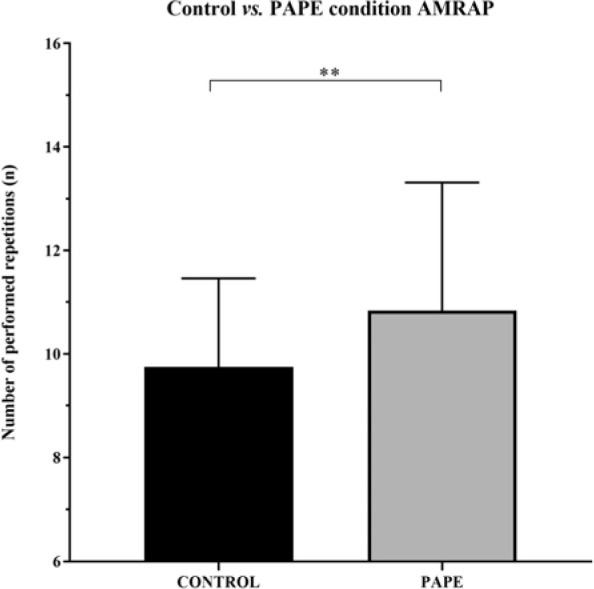
Total number of repetitions performed until volitional failure with 80% 1RM in the CON (control) and PAPE (experimental) conditions. ** p < 0.001

When comparing the number of repetitions performed until concentric volitional failure in a set with 80% of the 1RM, participants performed significantly more repetitions (10.83 ± 2.5 repetitions) under the PAPE condition than under the CON condition (9.76 ± 1.72 repetitions) (*p* = 0.008; ES = 0.5, small effect) (Figure 1).

**Figure 2:**
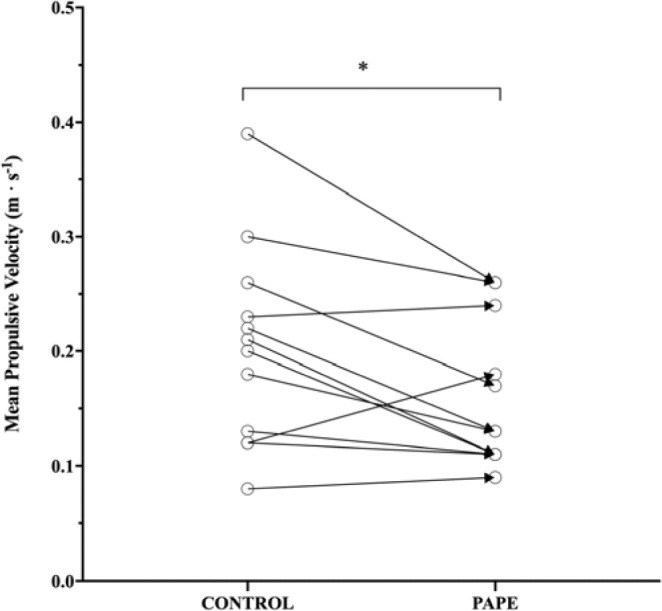
Mean propulsive velocity (m•s^-1^) of the last repetition of the performed set to volitional failure with 80% 1RM in the CON (control) and PAPE (experimental) conditions. * p < 0.05

The mean propulsive velocity of the last repetition prior to the concentric volitional failure was significantly lower under the PAPE condition (0.16 ± 0.06 m•s^-1^) than the CON condition (0.2 ± 0.09 m•s^-1^) (*p* = 0.02; ES = 0.52, small effect) (Figure 2). No differences were found in mean propulsive velocity of the first repetition between the PAPE (0.43 ± 0.1 m•s^-1^) and the CON condition (0.42 ± 0.13 m•s^-1^) (*p* = 0.582; ES = 0.09, small effect). Velocity loss from the first to the last repetition was significantly greater under the PAPE condition (0.27 ± 0.05 m•s^-1^) compared to the CON condition (0.22 ± 0.08 m•s^-1^) (*p* = 0.004; ES = 0.75, moderate effect).

The average reported RPE under the PAPE condition for the conditioning activity was 7.17 ± 0.58, and the achieved mean propulsive velocity in the conditioning activity was 0.30 ± 0.12 m•s^-1^.

## Discussion

The main finding of this study was that performing a traditional PAPE protocol consisting of a single set of a single repetition with 93% 1RM (Garbisu-Hualde and Santos-Concejero, 2021) improved bench press performance, measured as the number of repetitions performed to volitional failure. These results are in agreement with previous research (Alves et al., 2019).

Performing a traditional PAPE protocol prior to a set to volitional failure could make the athlete acutely more resistant to fatigue in long-lasting tasks (in our case, a set to volitional failure of various repetitions) (Bompa and Buzzichelli, 2016). The exact rationale behind this improvement in performance is not fully elucidated. Temperature increases are a commonly mentioned mechanism behind performance enhancement, but based on the results of Weigert et al. (2018), where small and non-significant temperature changes were seen after 10 repetitions at 70% in a biceps curl, it seems unlikely. Nevertheless, Boullosa et al. (2018) mention a possible mechanism, where the elevation of Ca^2+^ levels increases due to low-frequency twitches, what can cause the performance enhancement independent of myosin regulatory light chain phosphorylation.

Interestingly, performance improvements observed in this study may be related to improved capacity to perform slower repetitions, as under the PAPE condition, participants performed, on average, one more repetition, which was slower than under the CON condition (Figure 2). In consequence, the velocity loss was greater from the first to the last repetition. The attempt to complete one more repetition so close to failure could be due to psychological reasons (Szalma and Hancock, 2011). The maximal adaptability theory (Szalma and Hancock, 2011) states that hyperstress situations could lead to bad performance. In this way, those last, hard, and slow repetitions would be the stressing situations where participants need to strive to fulfil the lift. Performing the conditioning activity in the PAPE protocol (a heavy repetition prior to the tested task), could improve participants´ confidence when struggling with those last repetitions (Szalma and Hancock, 2011). Another possible explanation for the performance improvement observed could be the training velocity specificity, which means that after performing that specific conditioning activity, participants gain acute fitness or adapt acutely to low velocity lifting (Behm and Sale, 1993).

The quantity of work performed per session, understood as the total number of sets (Baz-Valle et al., 2022) or the volume load (sets x repetitions x kilogram) (Schoenfeld et al., 2014), is related to the quantity of gained muscle mass. Thus, if PAPE leads to an increased number of repetitions performed until failure, the volume load per session would be improved, and so could muscle hypertrophy. Following this line of reasoning, it could also be assumed that hypothetic muscle hypertrophy benefits could be due to both the improved mechanical tension of the last repetition (due to obtained lower mean propulsive velocities, Figure 2) and a greater number of performed repetitions (Figure 1). This means that the performed additional repetition may be effective when aiming at muscle hypertrophy. Previous studies support the notion that with a greater velocity loss, muscle hypertrophy gains can be more significant (Pareja-Blanco et al., 2017), but only to some extent (Andersen et al., 2021; Pareja-Blanco et al., 2020). Evidence suggests that when velocity loss is excessive (40%), subsequent sets could be affected (Pareja-Blanco et al., 2020). This is in line with findings of Alves et al. (2019), where significant differences were found in the number of repetitions performed in the first (PAP = 11.5 ± 3.1; CON = 10.4 ± 2.7; *p* < 0.05; ES = 0.38) and the second (PAP = 6.5 ± 1.9; CON = 5.5 ± 1.8; *p* < 0.05; ES = 0.54) set between PAP and CON groups, but not in the third set. This suggests that performance enhancement can increase training volume significantly, but when velocity loss is too pronounced, fatigue may overcome potentiation and impair performance in subsequent sets. Our study is in line with that by Krzysztofik et al. (2020b), as increasing the number of repetitions led to higher time under tension. Based on this, future research should address whether a group performing a PAPE protocol combined with a moderate velocity loss (i.e., 20% of velocity loss) can complete more repetitions than a control group for several sets (four to six sets). Furthermore, if the PAPE experimental condition group can perform more repetitions, it would be interesting to carry out a long-term intervention to examine whether this protocol would bring more muscle mass gain than a control condition.

This study faced several limitations, including a relatively small (*n* = 12) sample size, which makes it difficult to generalise the obtained results. Our results prove that a PAPE protocol can be useful to improve performance in a task to failure, which makes PAPE a potential strategy to increase muscle hypertrophy gains. However, this was not measured, and therefore, further studies are warranted. Furthermore, our study included only one set until volitional failure, however, the effect of this set on performance in subsequent sets was not evaluated.

## Conclusions

In conclusion, the results of this study suggest that a traditional PAPE protocol consisting of a single set of a single high intensity repetition (93% 1RM) improves bench press performance, measured as the number of repetitions completed until volitional failure. This could be due to psychological (Szalma and Hancock, 2011) or training specificity reasons (Behm and Sale, 1993). Also, performing a traditional PAPE augments velocity loss from the start of the set until the end, due to a greater capacity to perform slower last repetitions.
